# Hydrothermal extraction and physicochemical characterization of biogenic hydroxyapatite nanoparticles from buffalo waste bones for in vivo xenograft in experimental rats

**DOI:** 10.1038/s41598-023-43989-9

**Published:** 2023-10-15

**Authors:** Shada A. Alsharif, Mahmoud I. Badran, Moustafa H. Moustafa, Radwa A. Meshref, Ehab I. Mohamed

**Affiliations:** 1 University College in Umluj, Tabuk University, Tabuk, Kingdom of Saudi Arabia; 2 Medical Biophysics Department, Medical Research Institute, Alexandria University, 165 El-Horreya Avenue, Alexandria, 21561 Egypt; 3 Medical Equipment Technology Department, Faculty of Applied Health Sciences Technology, Pharos University, Alexandria, Egypt

**Keywords:** Biophysics, Medical research, Materials science

## Abstract

Hydroxyapatite (HA) can be used in odontology and orthopedic grafts to restore damaged bone due to its stable chemical characteristics, composition, and crystal structural affinity for human bone. A three-step hydrothermal method was used for the extraction of biogenic calcined HA from the buffalo waste bones at 700 °C (HA-700) and 1000 °C (HA-1000). Extracts were analyzed by thermogravimetric analysis, differential scanning calorimetry, X-ray fluorescence, X-ray diffraction, Fourier transform infrared spectroscopy, scanning electron microscopy, and in vivo examination of HA xenografts for femoral lesions in experimental rats. Crystallinity, purity, and morphology patterns showed that the HA main phase purity was 84.68% for HA-700 and 88.99% for HA-1000. Spherical HA nanoparticles were present for calcined HA-700 samples in the range 57–423 nm. Rats with critical bone lesions of 3 mm in diameter in the left femur treated with calcined HA-700 nanoparticles healed significantly (*p* < 0.001) faster than rats treated with HA-1000 or negative controls. These findings showed that spherical biogenic HA-700 NPs with a bud-like structure have the potential to stimulate both osteoconduction and bone remodeling, leading to greater bone formation potential in vivo. Thus, the calcined biogenic HA generated from buffalo waste bones may be a practical tool for biomedical applications.

## Introduction

Tissue and organ failure are critical concerns associated with longevity and a higher life expectancy due to increasing health expenditure and the continuous improvement of healthcare systems worldwide^1^. Tissue engineering involves creating, repairing, or replacing tissues and organs using biomaterials and biologically active molecules to restore, support, enhance function, or improve the quality of life of the patient^2,3^. Bioactive hydroxyapatite (HA), a calcium phosphate mineral, has been shown to stimulate bone restoration in both allografts and xenografts because of its stable chemical properties, composition, and crystal structural affinity for human bone^4,5^. However, hip and knee joint implants may fail due to many factors, including adverse body reactions, infection, pain, insufficient bonding, high stiffness, wear, improper placement, fractures, extensor mechanism failure, and aseptic loosening^6^.

HA can be chemically synthesized using calcium and phosphorus chemical precursors, yet researchers have investigated and extracted biogenic HA from a variety of natural sources, primarily from biogenic mammalian resources like bovine, dromedary, porcine, and birds^7–9^. HA can also be extracted from marine or aquatic sources, such as fish scales and bones, shell sources, such as cockles, clams, eggs, and seashells, plants, algae, and mineral sources (e.g., limestone)^10^. These natural substrates have beneficial mechanical properties, biocompatibility, and biodegradability, making them affordable, nontoxic, and suitable for various biomedical applications^4–6^. Additionally, these unique chemical formulations could enhance the adsorptive treatment of heavy metal and dye contaminated wastewater in environmental applications^11^. Calcination, alkaline hydrolysis, precipitation, and hydrothermal are among the techniques employed to extract biogenic HA for biomedical applications^5,12,13^. However, the characteristics of biogenic HA, such as, calcium-to-phosphorus ratio, crystallinity, phase structure, particle sizes, and morphology, are highly dependent on the extraction method^7,10,14^.

In the hydrothermal technique, the lysate of a substance is recrystallized at high temperature in a pressure autoclave to produce convection in the supersaturated state formation^13,14^. This procedure enables the extraction of highly-crystalline HA nanoparticles (NPs) from animal waste bones and calcinating them at high temperatures (normally in the range 600–1400 °C) to completely remove the organic matter and provide sterilized extracts^5–10,12–15^.

X-ray diffraction (XRD) patterns of defatted, deproteinized, and calcined mammalian bones from humans, porcines, and bovines showed ordered crystal structures in the nanoscale range. The XRD patterns for ordered crystals are determined by the crystal size, with broad peaks for biogenic HA NPs extracts due to elastic and inelastic contributions^16^. Moreover, there exists a negative association between particle-specific surface area and temperature and a positive correlation with HA particle sizes and temperature, which may be the result of crystallization and agglomeration^10^. Furthermore, the biogenic HA extracts, unlike synthetic HA, have trace embedded impurities (e.g., Na^+^, K^+^, Zn^2+^, Mg^2+^, Si^2+^, Ba^2+^, F^−^, $PO_{4}^{3 - }$, and $CO_{3}^{2 - }$) in the non-stoichiometric phase of the lattice, essential in xenograft for bone osteogenesis and remodeling in humans^10,15,16^. HA NPs are successfully used in biomedical realms, with viable implications in odontology and orthopedic grafts for fractured or damaged bone brought on by trauma, cancer, congenital, or other bone-related disorders^12,13^. Investigating the biological efficacy of biogenic HA as filling materials for critical size bone defect repair is still necessary as of this writing.

The aim of this study was to extract calcined biogenic HA from buffalo waste bones by a three-step hydrothermal approach, investigate its physicochemical properties, and evaluate its in vivo efficacy for treating critical-sized femoral lesions in experimental rats.

## Materials and methods

### Extraction of sterilized calcined buffalo HA powder

Buffalo (Bubalus *bubalis*) femur bones were obtained from healthy, mature sacrificed animals of three years old, sourced from the local slaughterhouse. The as-received bones from the slaughterhouse were thoroughly washed and cleaned at 100 °C (1:5, *w/v*, bone:water) for an hour to ease the removal of any organic substance from the bones^14,17^. 500 g of the clean bones were crushed into small pieces and placed in a 110 × 90 mm cylindrical autoclave at 130 °C and 1.5 atmo for 4 h to ease the removal of any organic residues and bone milling to achieve nanosized particles. The sterilized bones were further pulverized and sieved to a fine powder of uniform particle size. Finally, the raw bone powder was calcined and subjected to heat treatment in an electric box furnace under atmospheric conditions every 15 °C, till 700 and 1000 °C, at a heating rate of 5 °C/min and sintering for 2 h^17^. All samples were dried and labeled as raw bone (B-raw), calcined bone at 700 °C (HA-700), and calcined bone at 1000 °C (HA-1000) for subsequent in vivo testing on experimental rats.

### Characterization of calcined buffalo HA extracts

#### Thermogravimetric analysis and differential scanning calorimetry (TGA/DSC)

The thermal breakdown and crystallization behavior of B-raw bone powders were studied from 25 to 900 °C in a nitrogen environment at a heating rate of 15 °C/min using a thermal analysis instrument (SDT Q600DSC-TGA, USA)^18–24^. Thermogravimetric analysis (TGA) was employed to record the weight loss changes in bone powder with increasing temperature. Differential scanning calorimetry (DSC) was used to quantitatively and qualitatively describe the physicochemical characteristics induced by endothermic and exothermic processes in bone powders as the temperature changed.

#### X-ray diffraction (XRD) analysis

An X-ray diffractometer (GNR, APD 2000 Pro, Italy) with a CuK target (*λ* = 1.5406 Å) and a voltage and current setting of 40 kV and 30 mA was used to examine the crystalline structure of all three HA powders, as described earlier^18,25,26^. Analysis of the XRD patterns of B-raw, HA-700, and HA-1000 samples was performed in the range 2*θ* = 10°–80° with a step size of 0.05°. The standard diffraction data of the Joint Committee on Powder Diffraction Standards (JCPDS) reference 00-009-0432 for the HA phase was used for phase identification^28^, while the crystallite size (*X*_*c*_, nm) of the HA samples was calculated using Scherrer’s equation:^29^

1 $$X_{c} = k\lambda /\beta Cos\theta$$

where *k* is a constant equal to 0.9, *λ* is the X-ray wavelength (1.54 Å), *β* is the full width at half maximum (FWHM, radians), and *θ* is the diffraction angle of the reflection plane at [002].

The sample crystallinity (*D*_*c*_) was calculated using the Landi equation:^18^

2 $$D_{c} = 1 - V_{112/300} /I_{300}$$

where *D*_*c*_ is the crystallinity, *V*_112/300_ is the intensity of the hollow between (112) and (300) peaks, and* I*_300_ is the intensity of the (300) diffraction peak of the biogenic HA powders.

#### X-ray fluorescence (XRF) spectroscopy analysis

A hand-held X-ray fluorescence (XRF) analyzer (Delta Premium, Olympus, Waltham, MA, USA), which can detect elements from ^12^Mg through ^83^Bi, was used to quantify elemental composition of B-raw buffalo femur bone, as previously described^23,27–29^. The XRF was calibrated with a standard reference material before any measurements were made, and quality control was monitored using software^28^. Mineral content of B-raw samples was measured by scanning three locations on the femur midshaft for two minutes each. The percentage of elements that has measurement errors less than 5%, converted to mg/g units, was calculated by dividing the peak area of each element by the total area of all elements in the scan^27^.

#### Fourier transform infrared (FTIR) spectroscopy analysis

The functional spectral groups for B-raw, HA-700, and HA-1000 powders were determined through Fourier-transform infrared (FTIR) spectroscopy using a Bruker tensor 27 IR spectrometer (Bruker Optik GmbH, Ettlingen, Deutschland), as detailed elsewhere^16,30–32^. The spectra were studied in the transmittance mode at a spectral resolution of 4/cm over the wave number range of 400–4000/cm.

#### Scanning electron microscopy (SEM)

The surface morphology and microstructure of B-raw, HA-700, and HA-1000 samples coated with a sputter coating evaporator (model: SPI Module, Sputter Carbon/Gold Coater) were analyzed using scanning electron microscopy (SEM; JEOL JSM 6510 lv, Japan) at 30 kV acceleration voltage^33,34^.

### In vivo study on experimental animals

#### Experimental design

Twelve adult albino rats were unilaterally lesioned at the left femur bone using a motorized drill and split into three equal experimental groups (*n* = 4). The animals received sham and xenograft HA treatments for 4 weeks. Negative controls with non-treated femoral lesions made up Group I; animals treated with HA-700 for their bone lesions made up Group II; and animals treated with HA-1000 for their bone lesions made up Group III.

#### Animals

Albino rats (*n* = 12) of two-month age and weighing 220–250 g at the start of the experiment were randomly divided into three equal groups (*n* = 4) with ad libitum drinking water and were kept on standard chow. The rats were housed in large rectangular cages (49 × 34 × 16 cm), each of which could house up to 4 adult rats, and each group was allowed to acclimate to the natural daylight cycle for one week. The Institutional Animal Care and Use Committee (IACUC #0121961521) approved the study’s protocol before it began to ensure it followed the US National Institutes of Health’s Guide for the Care and Use of Laboratory Animals (NIH publication no. 85-23, revised 1996)^35^. In addition, the procedures used and described here are consistent with the standards set forth by ARRIVE guidelines. The Ethics Committee of the Medical Research Institute, Alexandria University, Alexandria, Egypt, approved the study protocol.

#### Surgical procedure

**Figure 1:**
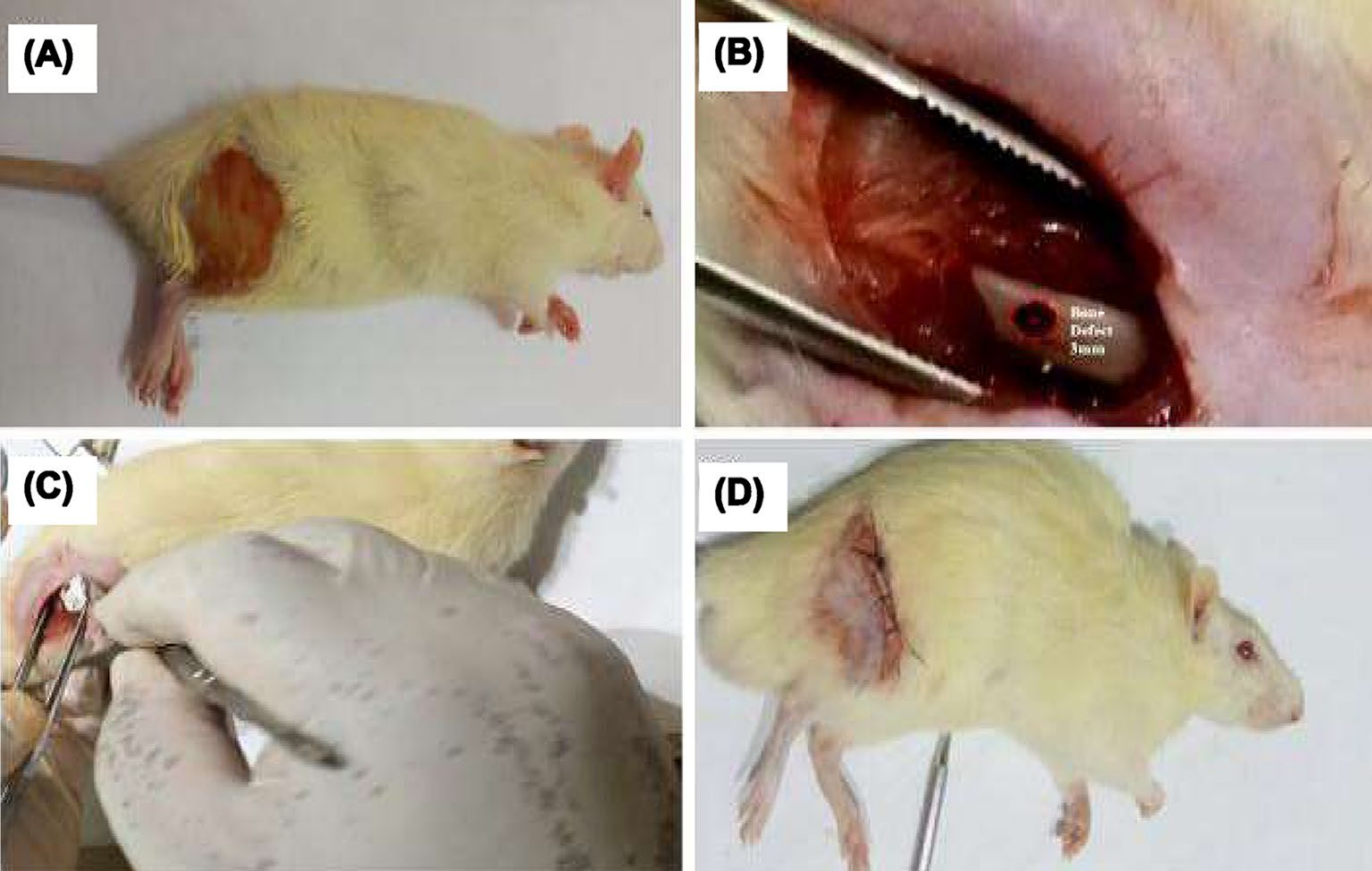
Surgical procedure showing an anesthetized rat with left femur shaved (**A**), periosteum and soft tissues fixed, and a 3-mm-diameter lesion drilled into femur bone to medullary canal (**B**), xenograft treatment with HA-700 or HA-1000 used to fill femur bone lesion (**C**) and wound closed with 5-0 suture (**D**).

Rats were anesthetized by injecting ketamine (50 mg/kg) and Lidocaine HCl (20 mg/ml) *i.p.* After the left femur was shaved, the periosteum and soft tissues were fixed, and a motorized drill was used to create a 3-mm-diameter lesion in the femur bone all the way to the medullary canal (as shown in Fig. 1A and B). Afterwards, xenograft treatment with HA-700 or HA-1000 was used to fill the femur bone lesion (Fig. 1C). After repositioning the skin and muscles, a 5-0 suture was used to close the wound (Fig. 1D).

#### Histology

After 4 weeks of sham and xenograft HA treatment, the rats were anesthetized once again, and a segment of the bone lesion region was removed from the left femurs. Afterwards, longitudinal serial sections 4–5 μm thick were cut from the samples with a microtome. The samples were then put in a 10% buffered formaldehyde solution for 24 h, and then they were decalcified in a 10% EDTA solution. Hematoxylin and Eosin (H&E) staining was used to analyze tissue sections under the microscope^36,37^.

#### Blinding and masking

Four different researchers worked on each animal as follows: one researcher (MIB) assigned the animals to treatment groups and administered the sham and xenograft HA treatments following a randomization table. This researcher was the only one who knew which group received which treatment. A second investigator (MHM) administered the anesthesia, and a third (SAA, MIB, MHM) performed the surgery. The lesions and histological findings in the femur bones were finally evaluated by a fourth investigator (RAM, EIM), who was also blinded to the treatment.

#### Outcome measurements

After 4 weeks of sham and xenograft HA treatment, all the animals showed signs of healing, which was measured both in terms of the size of the inflicted lesion on the femur and its histological appearance under the microscope.

#### Statistical analysis

An analysis of variance (ANOVA) followed by Tukey’s post hoc test of significance was used to compare the size of femur lesions between groups using the SPSS statistical package (Version 16; Chicago, IL, USA). The results were displayed in the form of means and standard deviations (SD). A *p*-value less than 0.05 was taken to show statistical significance.

## Results and discussion

The increasing annual demand for biogenic HA in healthcare is due primarily to the growing number of patients worldwide who require orthopedic treatment. In this study, biogenic HA was extracted from buffalo waste bones using a three-step hydrothermal method at varying temperatures. The following techniques were used to characterize and evaluate biogenic HA extracts for in vivo xenografts in experimental rats.

### TGA/DSC analysis

**Figure 2:**
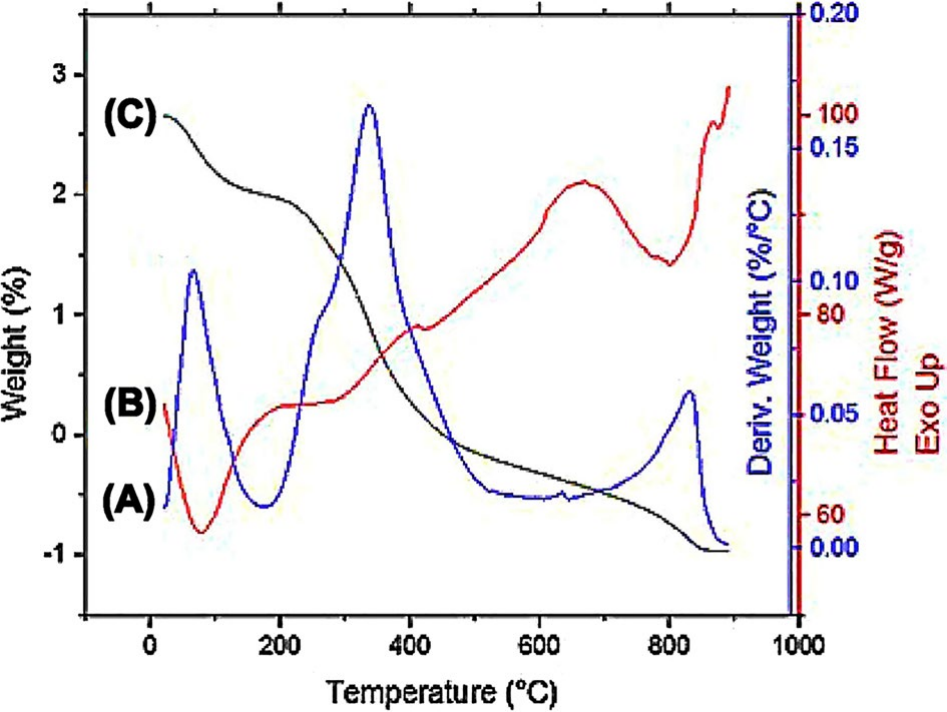
Derivative thermogravimetric analysis (DTGA) (**A**), thermogravimetric analysis (TGA) (**B**), and differential scanning calorimetry (DSC) (**C**) curves illustrating the temperature behavior for B-raw bone powder samples.

Derivative thermogravimetric analysis (DTGA), TGA, and DSC curves of B-raw bone powder sample decomposition due to temperature are shown in Fig. 2. Table 1 shows the DTGA temperature intervals, weight loss percentage, maximum decomposition peak temperature, and sample residues after combustion. Weight loss was initially seen in the range 38.66–169.57 °C of the DSC endothermic peaks due to the release of absorbed water and moisture content, accounting for 7.58% of the total mass with the maximum DTGA at 64 °C (Fig. 2A)^18,19^. A further 28.31% of the total mass is lost between 178.54 and 576.67 °C, which is characterized by a DTGA peak at 338.6 °C and a TGA sharp slope change (Fig. 2A and B), due to the combustion of bone’s organic components including collagen polymer fibrils, which triggers the exothermic HA recrystallization process^20–22^. The final weight loss seen in the range 585.63–883.33 °C, accounting for 7.27% of the total mass with a maximum DTGA at 830 °C (Fig. 2A), corresponds to the endothermic peak in the TGA curve at 700 °C (Fig. 2B), owing to the slow elimination of the $CO_{3}^{2 - }$ ions from the HA lattice^31,32^. These observations are consistent with those of the subsequent FTIR spectroscopy analysis, which confirms the crystallization of B-raw bone powders and the loss of $CO_{3}^{2 - }$ groups due to high temperatures.

**Table 1:** Temperature interval, maximum decomposition peak temperature, weight loss, and weight residue of bone powders by differential thermogravimetric analysis (DTGA).

Temperature interval (°C)	DTGA peak temperature (°C)	Weight loss (wt.%)			Residue (wt.%)
		*H*_2_*O*	Collagen	$CO_{3}^{2 - }$	
38.66–169.57	64.0	7.58			
178.54–576.67	338.6		28.31		56.39
585.63–883.33	830.0			7.27	

According to reports, thermal decarbonation commonly occurs between 400 and 600 °C in air and between 500 and 890 °C in a nitrogen environment^23^. No substantial weight loss related to the $CO_{3}^{2 - }$ decrease in the DTGA curve (Fig. 2A) was observed between 583 and 700 °C; this loss only becomes significant at 750 °C or higher^38^, reflecting thermal stability. In line with earlier studies, raw bone powder calcined at 700 °C gives the best thermal stability and avoids the sintering effect under drastic changes in temperature for the extraction of HA^37,39^. Further temperature increases from 830 to 1000 °C result in minimal weight loss (⁓ 0.45%, DSC curve Fig. 2C) suggesting that HA crystals have formed entirely at that point.

### XRD analysis

**Figure 3:**
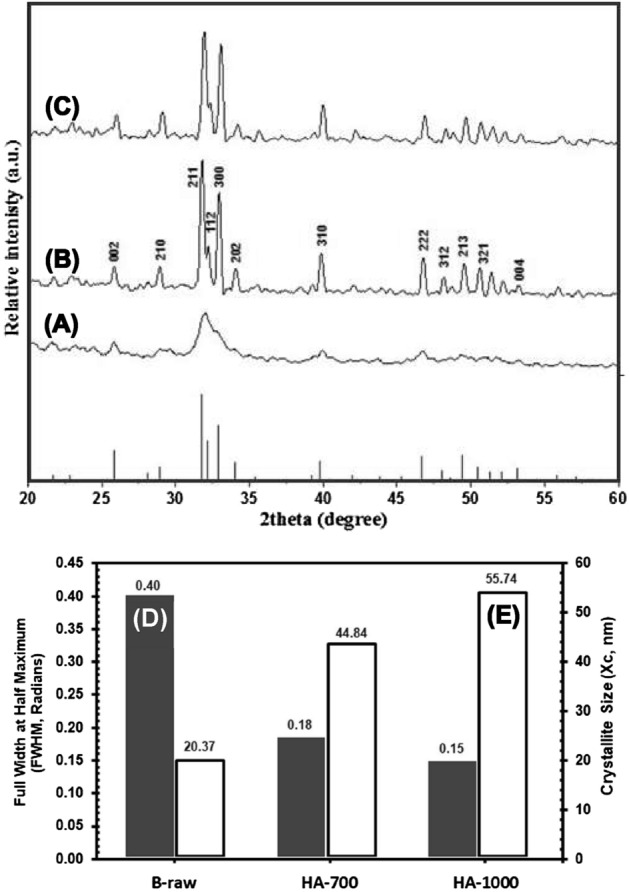
X-ray diffraction (XRD) patterns of B-raw (**A**), calcined HA-700 (**B**), and calcined HA-1000 (**C**) bone samples. Full width at half maximum (FWHM, radians) (**D**) and crystallite size (*X*_*c*_, nm) (**E**) at the diffraction peak 2*θ* = 25.78 corresponding to the reflection plane [002] for each of the three bone samples using Scherrer’s Eq. (1).

Crystallographic structure and phase purity of raw and calcined HA samples extracted at 700 °C and 1000 °C are shown by XRD patterns in Fig. 3. B-raw samples showed broad and poorly defined peaks at 2*θ* values of 25.85, 31.79, 39.83, and 46.74, respectively, which could not be reliably identified due to the presence of any leftover organic components as well as from the elastic and inelastic dispersion of the HA NPs crystals (Fig. 3A)^9,16^. The characteristic diffraction peaks of calcined HA-700 samples (Fig. 3B) found at 2*θ* = 25.78, 31.79, 32.21, 32.91, 34.01, 39.83, 46.74, 49.54, and 52.12, respectively, are well matched with the standard reference of the JCPDS 00-009-0432 for the HA phase^25^. The crystallite size (*X*_*c*_) of HA-700 samples was 44.84 nm, with an 84.68% crystallinity (*D*_*c*_), based on the diffraction peak at 2*θ* = 25.78 corresponding to the reflection plane [002]. Crystal size increased to 55.74 nm, and crystallinity increased to 88.99%, as shown by the narrower and sharper diffraction peaks of HA-1000 samples (Fig. 3C). In a similar earlier study, calcination was shown to increase the size of the crystals from nano to microscale and decrease the FWHM value, neither of which is directly associated with an improvement in crystalline quality^16,40^. Moreover, we did not observe any structural transformations or signs of secondary phases in the HA-700 and HA-1000 samples into beta-tricalcium phosphate (β-TCP) or calcium oxide (CaO), neither by hydrothermal treatment nor by calcination.

The FWHM and computed *X*_*c*_ at the diffraction peak 2*θ*= 25.78, which corresponds to the reflection plane [002], for B-raw, HA-700, and HA-1000 bone samples are shown in Fig. 3D and E. The higher FWHM value of 0.40 rad was associated with an *X*_*c*_ of 20.37 nm for B-raw samples. In similar studies, it was argued that the well-ordered and nanometric nature of biogenic HA nanocrystals from porcine origin resulted in broad XRD peaks, high FWHM values^16,40,41^, and crystallite size of comparable values (i.e., 22 nm)^42^. After the calcination, the values of FMWH dramatically decreased, which was responsible for an increase in the *X*_*c*_ for HA-700 and HA-1000 (i.e., 44.84 and 55.74 nm, respectively). Evidence from XRD peaks and a similar decrease in the FWHM suggests that crystal growth is initiated during the calcination process of biogenic bovine HA^43^.

### XRF spectroscopy analysis

B-raw bone elements with measurement errors of less than 5% among locations on the femur midshaft were selected and shown in Table 2: *Ca*, *Cu*, *Fe*, *Mg*, *Mn*, *P*, *K*, *S*, *Si*, *Zn*, and *Ca*/*P* ratio. The values of *Ca*, *Fe*, *Mn*, *P*, and *Zn* for buffalo femur B-raw bones agree well with those for buffalo humerus bones by Buddhachat et al.^27^, who employed the same technique for studying elemental composition in horns, teeth, and humeral bones of 14 different species, including humans, pigs, sheep, and buffalo. Moreover, the *Ca*/*P* ratio for buffalo femur B-raw bones was similar to that for humerus buffalo bones by the same group (i.e., 3.85 ± 0.05 vs. 3.70 ± 0.12, respectively)^27^. Furthermore, the values of *Ca*, *P*, *K*, and other bone minerals as well as the *Ca*/*P* ratio were similar to qualitative XRF elemental analysis in cow femur bones by Akindoyo et al.^23^, and recent findings by Yılmaz et al.^44^, in adult male and female guinea pigs.

**Table 2:** Elemental mineral content of buffalo B-raw femur bone by X-ray fluorescence (XRF) spectroscopy analysis.

Mineral	Concentration (mg/g)
Calcium (*Ca*)	239.46 ± 20.22
Copper (*Cu*)	0.04 ± 0.02
Iron (*Fe*)	3.65 ± 1.61
Magnesium (*Mg*)	24.14 ± 2.39
Manganese (*Mn*)	0.39 ± 0.25
Phosphorus (*P*)	62.34 ± 12.55
Potassium (*K*)	4.98 ± 1.58
Sulphur (*S*)	1.74 ± 0.46
Selenium (*Se*)	4.97 ± 0.83
Zinc (*Zn*)	0.28 ± 0.11

### FTIR spectroscopy analysis

**Figure 4:**
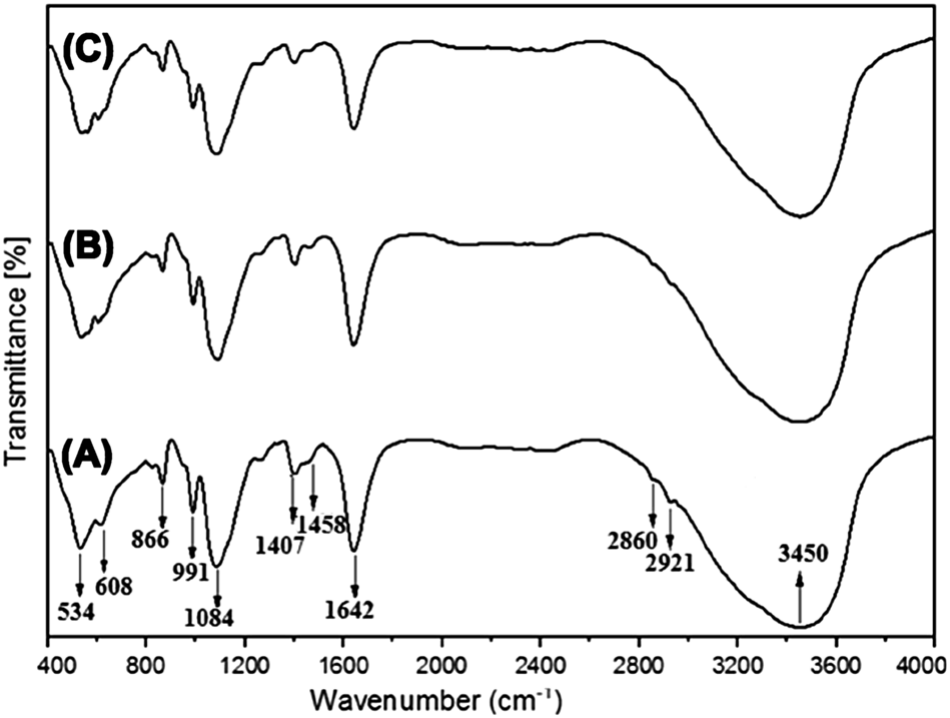
Fourier transform infrared (FTIR) analysis spectra of B-raw (**A**), calcined HA-700 (**B**), and calcined HA-1000 (**C**) bone samples.

The main functional ions $PO_{4}^{3 - }$, $CO_{3}^{2 - }$, and $OH^{ - }$, water molecules ($H_{2} O$), and organic matrix of extracted bone samples as detected by the FTIR spectroscopy, in comparison with earlier similar studies^16,45–51^, are shown in Table 3 and Fig. 4. B-raw samples showed a wide absorption band of organic components (collagen) at 2860.50 and 2921.43/cm (Fig. 4A), corresponding to the *C–H* symmetrical and asymmetrical stretching vibrational modes^16,50,51^. After calcination, $PO_{4}^{3 - }$ characteristic vibrational modes corresponding to the mineral bone component were present at 989.55/cm symmetric stretching (ν_1_)^46^, 1089.63/cm asymmetric stretching (ν_3_)^45,46,48^, 561.09, and bending 607.58/cm (ν_4_)^48^, as shown in Fig. 4B. The $CO_{3}^{2 - }$ group was present in and outside the HA-700 lattice, with characteristic spectral bands in the region 1404–1458/cm due to the doubly generated asymmetric stretching vibration (ν_3_)^45,46,48,49^. The peak at 866.15/cm is believed to be due to the non-apatite environment’s out-of-plane bending mode (ν_2_)^45,49^, which confirms the carbonation of HA-700 samples^31^. The $CO_{3}^{2 - }$ ions present in the lattice structure of HA-700 are known to be medically preferred because they enhance its biodegradability^2–6,12,13,17,19,22,24,36^. Adsorbed *H*_2_*O* was conformed from the broad band centered at 3450.77/cm and an intense band at 1639.98/cm for HA-700 and at 3452.48/cm and 1639.90/cm for HA-1000 calcined samples (Fig. 4B and C), which are always present in bone-derived biological apatite^32,48,50^. Moreover, the characteristic stretching mode of the $OH^{ - }$ group faded in the spectra for all sintered samples due to overlap with the broad *H*_2_*O* absorption band centered at 3450/cm, also attributed to an increase in $CO_{3}^{2 - }$ substitution. Furthermore, calcined HA-1000 (Fig. 4C) showed a shift to lower wavelengths in the characteristic $PO_{4}^{3 - }$ group’s band at 559.34, 605.63, and 1087.82/cm for the *v*_3_ and *v*_4_ vibrational modes^16,23,35–37^. Besides, the absorption bands at 1404–1460/cm of ν_3_ (*CO*_*3*_)^*2*^ of reduced intensity showed that HA might lose $CO_{3}^{2 - }$ groups due to high temperatures up to 1000 °C^16,45,46,48,49^.

**Table 3:** Band positions for B-raw, HA-700, and HA-1000 bone samples and reference band positions of earlier studies by Fourier transform infrared (FTIR) spectroscopy analysis.

Functional group	Vibration mode	Wavenumber (/cm)			Reference wavenumber (/cm)	References
		B-Raw	HA-700	HA-1000		
$PO_{4}^{3 - }$	Bending ν_4_ of $PO_{4}^{3 - }$ group	[608.32]	[561.09] [607.58]	[559.34] [605.63]	561	^45^
					562, 600	^16^
					565, 603	^46^
					568	^47^
					601	^48^
	Asymmetric stretching ν_3_ of $PO_{4}^{3 - }$ group	[1084.37]	[1089.63]	[1087.82]	1043, 1091	^46^
					1087	^45^
					1091	^48^
	Symmetric stretching ν_1_ of $PO_{4}^{3 - }$ group	[991.28]	[989.55]	[989.45]	961	^45^
					964	^46^
					993	^46^
$CO_{3}^{2 - }$	Asymmetric stretching ν_3_ of $CO_{3}^{2 - }$ group	[1407.74–1458.48]	[1404.00–1458.00]	1404.14–1460	1408–4158	^49^
					1450	^45^
					1459	^48^
					1458	^46^
	Out of plane bending ν_2_ of $CO_{3}^{2 - }$ group	[866.15]	[865.64]	[866.00]	868	^49^
					878	^45^
$OH^{ - }$	Bending mode of *H*_2_*O* molecule	[1642.82]	[1639.98]	[1639.90]	1642	^50^
	Stretching vibrations of *O*–*H* bonds in adsorbed water molecules	[3448.92]	[3450.77]	[3452.48]	3419	^48^
*Collagen*	*C*–*H* and *N*–*H* stretching modes	[2860.50] [2921.43]	–	–	2800–3400	^51^
					2854, 2925	^16^
					2885, 2925	^50^

### SEM analysis

**Figure 5:**
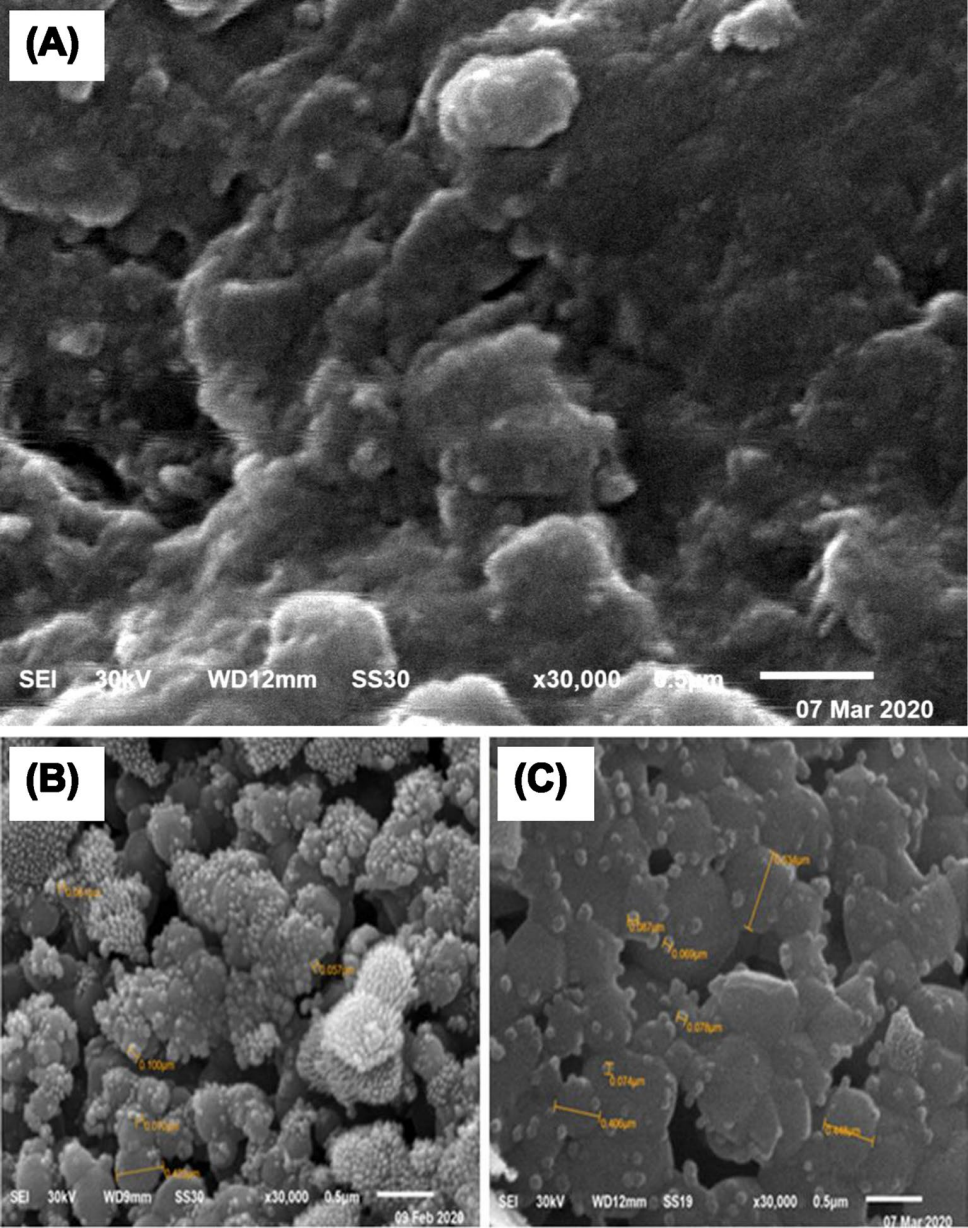
Scanning electron micrographs (SEM) of B-raw (**A**), calcined HA-700 (**B**), and calcined HA-1000 (**C**) bone samples at a standard magnification of 30,000×.

The SEM images of surface morphology for the raw and calcinated bone powder samples at a standard magnification of 30,000× are shown in Fig. 5. Like earlier studies^33,34^, B-raw samples showed the presence of HA microparticles with an average size of 0.58 ± 0.47 μm of irregular shape and rough surface, together with organic components (Fig. 5A). Calcinated HA-700 samples showed a broad bud-like particle structure, with small spherical HA NPs of 57 nm on top of others of 423 nm size (Fig. 5B). Further higher calcination temperatures yielded aggregated particles of irregular shape in the range from 63 to 639 nm, as shown for HA-1000 samples in Fig. 5C. It has been pointed out earlier that after the calcination of raw bone powders at 700 °C, particles become more regular and spherical in shape^34^. However, the majority of the HA extracted from mammalian bone exhibits irregular shapes, with some investigations showing the existence of flakes, rods, needles, and plate-like shapes^52–56^.

During the calcination process, HA samples experience many changes, including crystallite size, shape, and crystalline quality, as well as dehydrogenation and Mg release at high temperatures^57^. It has recently been shown that the reduction in FWHM in biogenic bovine HA samples produced by controlled calcination at temperatures ranging from 400 to 720 °C is due to coalescence mechanisms directly related to crystallite size increase^58^. Furthermore, the XRF analysis in Table 2 shows that the B-raw bone samples have a significant concentration of Mg; thus, the nanosized spherical particles seen in SEM micrographs for the HA-700 and HA-1000 samples correspond to *MgO* in the calcined samples^42,58,59^.

The bud-like structure of HA-700 NPs of two different sizes is remarkably original and has never been seen previously. Therefore, we suggest using the bud-like HA-700 NPs to treat critical-sized bone lesions. In the section that follows, it will be shown how treating critical-sized femoral lesions in a rat experimental model with in vivo xenografts may affect the biodegradability of bone during bone healing.

### In vivo study on experimental animals

**Figure 6:**
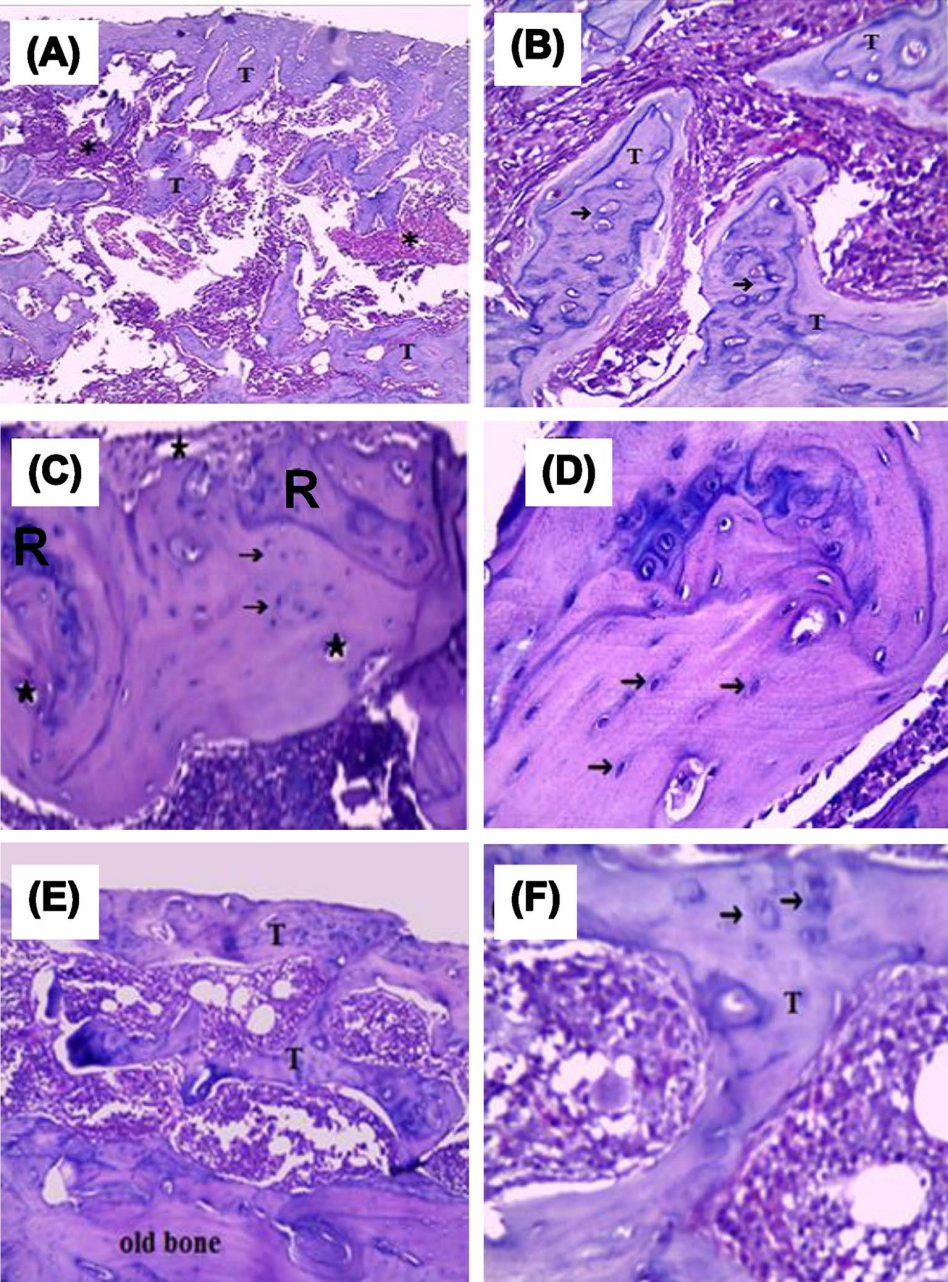
Photomicrographs of the left femur shaft of negative control animals (*n* = 4) showing large lesion sites filled with irregularly formed unevenly stained widely separated trabeculae of woven bone (T) with mild inflammatory cell infiltration (*) (**A**); unevenly stained bone trabeculae exhibiting larger and more irregularly shaped lacunae (black arrow) at a higher magnification (**B**); treated animals (*n* = 4) with HA-700 particles after four weeks showing lesions filled with new bone except for few small localized areas (*) and small islands of cartilage (R) (**C**); an acidophilic normal compact bone containing normal osteocytes inside their lacunae at a higher magnification (**D**); treated animals (*n* = 4) with HA-1000 particles after 4 weeks showing a few widely separated trabecular bone (T) with a persistence large lesion (**E**); and trabecular bone containing few osteocytes with irregular shape at a higher magnification (**F**). (H&E Original magnification **A**, **C**, and **E** 200×; **B**, **D**, and **F** 400×).

Photomicrographs at 200× of the left femur shaft of the negative control animals after four weeks showed large lesion sites filled with irregularly formed, unevenly stained, widely separated trabeculae of newly woven bone with mild inflammatory cell infiltration next to the lesion area (Fig. 6A). Unevenly stained bone trabeculae showing larger, densely grouped, and irregularly shaped osteocyte lacunae were also seen at 400× (Fig. 6B). Moreover, rat’s left femur shaft treated with calcined HA-700 NPs after the same period showed lesion sites filled with new bone except for a few small, localized areas, small islands of cartilage, and an acidophilic normal compact bone (Fig. 6C) with normal osteocytes inside their lacunae (Fig. 6D). There were no inflammatory or foreign body reactions when using spherical calcined HA-700 NPs, which were osteoconductive and exhibited early biosorption, according to previous recent investigations, demonstrating healthy bone repair^36,37,60,61^. These results also lend credence to in vivo investigations showing that the substitution of $CO_{3}^{2 - }$ ions in the HA-700 NPs’ lattice structure induces a favorable affinity for osteoblast cells, boosting cellular adhesion and collagen synthesis^62–65^. Furthermore, the rat’s left femur shaft treated with HA-1000 particles after four weeks showed few widely trabeculae of woven bone, with mineralized and unmineralized areas, at the upper part of the lesion, with a partial bridging bon at the center at 200× (Fig. 6E). A persistent large lesion was also seen at 400× with trabecular bone, including a few osteocytes of irregular shape (Fig. 6F).

Based on these observations, we deduce that calcined HA-700 NPs significantly (*p* < 0.001) accelerated the healing of experimental rats with critical bone lesions of 3 mm in diameter in the left femur when compared to HA-1000 or negative controls. It has been shown earlier that HA-1000 particles had lower $CO_{3}^{2 - }$ ion contents, high crystallinity, and a large crystal size^66,67^, which can explain their low resorption rate and poor integration with the newly formed woven bone in the lesion site during the healing process. Brandt et al.^68^, previously reported that after 12 weeks of nanocrystalline HA implantation for bone repair, rabbit femoral bones showed bone formation, low degradation, and slow resorption. Clinical studies showed that HA could form direct physiological bonds with bone, resulting in good biocompatibility and no inflammatory response^69^.

## Conclusion

An efficient three-step hydrothermal approach was proposed to extract the pure crystalline phase of biogenic HA from buffalo waste bones, which was found to be stable throughout the range of temperatures investigated. The optimal temperature for the production of crystalline HA in the bone structure was reported to be 700 °C, which was sufficient for eliminating residual organic matter and enhancing the crystallinity of the HA phase. HA-700 extracts were characterized by their bud-like nonstoichiometric wide nano-range particle size and the presence of divalent $PO_{4}^{3 - }$ and $CO_{3}^{2 - }$ anions on the non-apatite hydrated layer. Despite their limited biodegradability, HA-700 NPs could form bone-like apatite coatings on their surfaces for better bone bonding, which is important in bone remodeling for in vivo xenograft treatment of critical sized femoral lesions in an experimental rat model. Thus, the study confirmed the effectiveness of manufacturing porous biogenic HA bodies for biomedical applications from buffalo waste bones using the hydrothermal technique.

## Data Availability

Metadata used and/or analyzed during the current study will be made available from the corresponding author on reasonable request.
